# Sensing of an HIV-1–Derived Single-Stranded RNA-Oligonucleotide Induces Arginase 1-Mediated Tolerance

**DOI:** 10.3390/cells13131088

**Published:** 2024-06-23

**Authors:** Chiara Suvieri, Giada Mondanelli, Ciriana Orabona, Maria Teresa Pallotta, Eleonora Panfili, Sofia Rossini, Claudia Volpi, Maria Laura Belladonna

**Affiliations:** Section of Pharmacology, Department of Medicine and Surgery, University of Perugia, 06129 Perugia, Italy; chiara.suvieri@unipg.it (C.S.); giada.mondanelli@unipg.it (G.M.); ciriana.orabona@unipg.it (C.O.); maria.pallotta@unipg.it (M.T.P.); eleonora.panfili@unipg.it (E.P.); sofia.rossini@unipg.it (S.R.); claudia.volpi@unipg.it (C.V.)

**Keywords:** tolerance, RNA-oligonucleotides, small synthetic oligodeoxynucleotides, TLR3/TLR7 heterodimer, HIV-1

## Abstract

Small synthetic oligodeoxynucleotides (ODNs) can mimic microbial nucleic acids by interacting with receptor systems and promoting immunostimulatory activities. Nevertheless, some ODNs can act differently on the plasmacytoid dendritic cell (pDC) subset, shaping their immunoregulatory properties and rendering them suitable immunotherapeutic tools in several clinical settings for treating overwhelming immune responses. We designed HIV–1–derived, DNA- and RNA-based oligonucleotides (gag, pol, and U5 regions) and assessed their activity in conferring a tolerogenic phenotype to pDCs in skin test experiments. RNA-but not DNA-oligonucleotides are capable of inducing tolerogenic features in pDCs. Interestingly, sensing the HIV–1–derived single-stranded RNA-gag oligonucleotide (RNA-gag) requires both TLR3 and TLR7 and the engagement of the TRIF adaptor molecule. Moreover, the induction of a suppressive phenotype in pDCs by RNA-gag is contingent upon the induction and activation of the immunosuppressive enzyme Arginase 1. Thus, our data suggest that sensing of the synthetic RNA-gag oligonucleotide in pDCs can induce a suppressive phenotype in pDCs, a property rendering RNA-gag a potential tool for therapeutic strategies in allergies and autoimmune diseases.

## 1. Introduction

Dendritic cells (DCs) are highly versatile cells in terms of immunological behavior. Crucially, depending on the belonging subset, they can either direct the efficient priming of naïve T lymphocytes or maintain the tolerance toward self-antigens and immune homeostasis [1]. Traditionally, DCs have been classified into two major subpopulations based on developmental origin, surface markers, and functions. In particular, conventional DCs (cDCs) are specialized in antigen uptake and the presentation to naïve T-cells, whereas plasmacytoid DCs (pDCs) are typically activated during viral infections. However, pDCs can also play a variety of other functions, including T-cell stimulation and pro-inflammatory cytokine and chemokine secretion [1]. The rapid antiviral response of pDCs is triggered mainly by the endosomal sensors TLR3, TLR7, and TLR9, which recognize viral nucleic acids (dsRNA, ssRNA, and DNA, respectively) [2], and involves an abundant production of type I interferons (IFN-α and IFN-β) [1]. Nevertheless, the belief that these cytokines are endowed with an antiviral effect has been recently revised based on multiple pieces of evidence suggesting that prolonged type I IFN secretion rather suppresses the immune system and thus promotes viral persistence [3].

Recognition of unmethylated oligonucleotide sequences containing CpG motifs, particularly abundant in bacterial and viral nucleic acids, was identified as one of the major activation mechanisms of the DC immune response. Therefore, short synthetic oligodeoxynucleotides containing CpG sequences (CpG-ODNs) were used as vaccine adjuvants for the prevention/treatment of cancer and infectious diseases [4]. However, some studies have documented that CpG- and GpC-ODNs may also lead to the activation of indoleamine 2,3-dioxygenase 1 (IDO1)-dependent suppressive pathways, conferring tolerogenic properties to pDCs [5,6]. Thus, microbial-derived ODNs have been identified as potential therapeutic tools based on their dual opposite activities, either immunoadjuvant or tolerance-inducing.

DCs exploit multiple pathways to promote immune tolerance. The amino acid degrading enzymes Ido1 and Arginase 1 (Arg1) are major players in the induction of immunosuppressive responses by DCs [7]. Ido1 activates in these cells a tolerogenic program by multiple mechanisms, depending on its enzymatic and signaling functions [7]. Ido1-mediated L-tryptophan (Trp) catabolism produces immunoregulatory molecules downstream of L-kynurenine (Kyn), which can bind and activate the aryl hydrocarbon receptor (AhR), further inducing *Ido1* expression and generating a self-amplification circuit for the maintenance or the new onset of a stable regulatory phenotype in DCs [8]. Instead, the Ido1 signaling function, activated by TGF-β and dependent on the phosphorylation of two immunoreceptor tyrosine-based inhibitory motifs (ITIMs), induces the expression of *Tgfb* and *Ido1* themselves, thus establishing a feedforward amplification loop that confers a long-term immunoregulatory phenotype on both cDCs and pDCs [7,9,10]. On the other hand, Arg1 hydrolyzes L-arginine into urea and L-ornithine (Orn), a substrate of ornithine decarboxylase (ODC) for the generation of polyamine pathway catabolites. Polyamines produced downstream of *Arg1* induction in DCs and other cell types (for instance, myeloid-derived suppressor cells, MDSCs) can condition DCs to acquire an immunosuppressive phenotype through the activation of Src kinase, with Ido1 among its phosphorylation targets [9]. Thus, in immunometabolism, Arg1 and Ido1 are linked by mutual promoting pathways, and their activation could represent a fundamental tool for the induction and maintenance of a long-term tolerance state [9].

Even though antiretroviral therapy successfully suppresses human immunodeficiency virus (HIV) replication, current therapies are not efficient at eradicating the infection yet. The major barrier to complete clearance of the virus is the persistence of a latent reservoir in long-lived resting and proliferating memory CD4^+^ T-cells. Moreover, little is still known about how immune cells can sense and respond to HIV [11]. pDCs are among the first cells to encounter the virus after host invasion [12], and their role in HIV infection and acquired immunodeficiency syndrome (AIDS) development is both beneficial and detrimental at the same time. In fact, pDCs produce high levels of type I IFNs, inhibiting viral replication and inducing bystander T-cell activation. However, they can also release chemokines, such as CCL5, which, by recruiting CCR5^+^ CD4^+^ T-cells to the infection site, facilitate the viral spread. However, it is known that CCL5 competes with HIV-1 gp120 protein for CCR5 co-receptor binding, thus having an inhibitory effect on HIV-1 entry and replication [13]. It has also been shown that after in vitro stimulation with TLR7 or TLR9 ligands, pDCs from HIV-1-infected people have a decreased ability to generate type I IFNs [14,15]. Furthermore, increased blood levels of T-cell immunoglobulin and mucin domain-containing 3-positive (Tim-3^+^) exhausted pDCs and increased expression of CD40 on blood pDCs during HIV-1 infection have all been linked to decreased production of IFNα [15,16]. The majority of these studies used HIV-1 in-vitro-exposed pDCs obtained from healthy donors or patients in the HIV-1 chronic stage. On the other hand, the low but persistent production of type I IFNs during chronic HIV-1 infection can induce apoptosis of T-cells, thus contributing to a decreased CD4^+^ cell count. Finally, the expression of *Ido1* in pDCs skewing Treg/Th17 homeostasis in favor of immunosuppressive Treg cells [17] is another piece of the complex puzzle depicting pDC regulatory functions in viral infections.

In this paper, we show that the synthetic HIV-1–derived single-stranded RNA-gag oligonucleotide (RNA-gag) can start a tolerogenic program in pDCs through a so far unknown mechanism involving both TLR3 and TLR7, TRIF recruitment, and *Arg1* induction and activation. These findings could be crucial to understanding how pDCs recognize HIV and, more importantly, might provide a potential immunotherapeutic tool to shape the pDCs’ functions for the induction of immunosuppression in negative vaccination strategies against allergies and autoimmune diseases.

## 2. Materials and Methods

### 2.1. Mice and Reagents

Six-week-old female C57BL/6 mice were obtained from Charles River Laboratories (Calco, Lecco, Italy). Mice homozygous for the TLR3 targeted mutation (*Tlr3*^−/−^) raised on the C57BL/6 background were generated as described [5], and bred at the animal facility of the University of Perugia. Mice homozygous for the TLR7 mutation (*Tlr7*^−/−^) were obtained from The Jackson Laboratory (Bar Harbor, ME, USA).

The HY peptide (WMHHNMDLI) and endotoxin-free RNA- and DNA-oligonucleotides, listed in Table 1, all on a phosphorothioate backbone, were purchased from Biofab Research (Rome, Italy). Endotoxin contamination, as assessed by the Limulus amebocyte assay (Sigma Aldrich, St. Louis, MO, USA), was negligible in the preparations of all oligonucleotides.

### 2.2. DC Preparation, Treatment, and Transfection

All purification procedures for pDCs and cDCs have been previously described [7]. Briefly, splenic cDCs and pDCs fractions (CD11c^+^/CD8^−^ and CD11c^+^/mpDCA1^+^, respectively) were obtained by magnetic beads selection according to their specific markers (Miltenyi Biotec, Cologne, Germany), as previously described [6]. For all in vitro studies, 1 × 10^6^ cDCs or pDCs per well were cultured in 24-well plates in Iscove’s Modified Dulbecco’s medium (IMDM) (Thermo Fisher Scientific, Waltham, MA, USA). Purified pDCs were exposed for 24 h at 37 °C to different oligonucleotides at the concentration of 1.5 μM.

For *Ticam1*, *Myd88*, *Ido1*, or *Arg1* silencing, gene-specific small interfering RNAs (siRNA) were predesigned based on the gene sequence (catalog numbers s98709, s70237, 62191, and s62579, respectively) and were synthesized by Ambion Life Technologies (Carlsbad, CA, USA). Transfection of pDCs was performed as previously described [10]. Briefly, siRNAs (6.7 μg, corresponding to 1 nmole) in 30 μL transfection buffer (20 mM HEPES, 150 mM NaCl, pH = 7.4) were gently mixed with 6.7 μg 1,2-dioleoyl-3-trimethylammonium-propane (DOTAP, Merck, Darmstadt, Germany) in 30 μL transfection buffer. After incubation at room temperature for 20 min, the mixture was added to 1 mL complete medium containing 1 × 10^6^ pDCs and incubated for 24 h at 37 °C. Cells were then recovered, washed, and immediately used for in vivo experiments. DNA- and RNA-oligonucleotides were added 6 h after the silencing procedure.

### 2.3. Skin Test Assay

A skin test assay was used to evaluate the major histocompatibility complex class I–restricted delayed-type hypersensitivity (DTH) response to the HY peptide (i.e., male minor antigen) in C57BL/6 female recipient mice [18]. In DTH experiments, each group is composed of 6 randomized inbred mice, not requiring specific inclusion or exclusion criteria, housed in a single cage, and provided with food and water. N = 6 for each group was decided based on previous DTH experimental results revealing that this number of mice per group can give an alpha of 0.05 and a power of 0.80.

For immunization, cells were loaded with peptide (5 mM, 2 h at 37 °C) and i.p. injected into recipient hosts (n = 6 per group). A total of 3 × 10^5^ peptide-pulsed cDCs (majority fraction) were injected either alone or in combination with a minority fraction (5%, i.e., 1.5 × 10^4^ cells) of pDCs purified from spleens of wild-type, *Tlr3*^−/−^, or *Tlr7*^−/−^ mice, and overnight incubated without (untreated control) or with 1.5 μM of DNA- or RNA-oligonucleotides. Two weeks after immunization, the DTH response to the intrafootpad challenge with the eliciting peptide was measured, and the difference between the left hind receiving the challenging peptide and the right hind receiving vehicle alone for each mouse was calculated. Hairline cutting and hind weighting were performed in a blind manner, as the operator was unaware of the group treatment. Results were expressed as the footpad weight increase of peptide-injected footpads over that of the vehicle-injected counterparts. In the reported DTH experiments, an internal control is used for each animal. Within each group, the values of footpad weight increase are used to calculate the mean, standard deviation, and P values shown in the bar graph. For each group, a P value equal to or below 0.05 reveals a significant footpad weight increase, which is a significant immunogenic response. Therefore, in the DTH experiments, rather than the significant change between an experimental group and a control group, the eventual significant weight increase that occurred within a certain group is statistically analyzed because it is an all-or-nothing readout for the activation (significant footpad weight increase) or not (non-significant footpad weight increase) of an immunogenic response against the challenging peptide. A treatment that abrogates an otherwise activated immunogenic response can be considered capable of inducing tolerance.

### 2.4. Cell Lines and Immunoprecipitation

For co-immunoprecipitation, cells expressing *Tlr7-HA* and/or *Tlr3-Myc* were obtained by electroporation of 1 × 10^7^ HEK293 cells (ATCC, Manassas, VA, USA) with 20 µg of plasmid DNA containing the gene construct of interest. Briefly, the used plasmids were pUNO, expressing the murine *Tlr7* gene fused at the 3′ end to the influenza hemagglutinin (HA) (Invivogen, San Diego, CA, USA), or pCMV6 containing the murine *Tlr3* gene fused at the 3′ end to the *Myc* tag (Origene, Rockville, MD, USA). Transfected cells were lysed on ice in Lysis buffer (20 mM Tris-HCl, pH = 7.4, 50 mM NaCl, 1% TRITON X-100 and protease inhibitors). Lysates were first immunoprecipitated using a mouse monoclonal antibody specifically recognizing HA (2-2.2.14 clone, Thermo Fisher Scientific, MA, USA) and then incubated with protein A-agarose (Sigma-Aldrich, St. Louis, MO, USA). Samples incubated with protein A-agarose alone were used as controls. Immunoprecipitated proteins were analyzed by immunoblot with the same anti-HA and anti-Myc (9E10 clone, Origene, Rockville, MD, USA) antibodies, in combination with appropriate horseradish peroxidase-conjugated antibody (Merk Millipore, Burlington, MA, USA), followed by enhanced chemiluminescence (ECL, Bio-Rad, Hercules, CA, USA).

### 2.5. Real-Time PCR and Determination of Arg1 and Ido1 Catalytic Activity

Real-time PCR for mouse *Ido1*, *Arg1*, and *Gapdh* analysis was carried out as described [7], using the following primers (Biofab Research, Rome, Italy): *Ido1* forward primer 5′-CGATGTTCGAAAGGTGCTGC-3′; *Ido1* reverse primer 5′-GCAGGAGAAGCTGCGATTTC-3′; *Arg1* forward primer 5′-CAGAAGAATGGAAGAGTCAG-3′; *Arg1* reverse primer 5′-CAGATATGCAGGGAGTCACC-3′; *Gapdh* forward primer 5′-TGCCCAGAACATCATCCCT-3′; *Gapdh* reverse primer 5′-ACTTGGCAGGTTTCTCCAGG-3′. Data were calculated as the ratio of the gene to *Gapdh* expression by the relative quantification method (ΔΔCT; means ± SD of triplicate determination), and data are presented as normalized transcript expression in the samples relative to normalized transcript expression in control cultures.

Arg1 activity was measured in cell lysates from 24-h cultures in terms of urea production, as described [9]. The urea concentration was determined by measuring absorbance at 430 nm using a UV/visible spectrophotometer (TECAN, Thermo Fisher Scientific, Waltham, MA, USA) and then normalized for the total protein content. Ido1 enzymatic activity was evaluated in terms of its ability to metabolize Trp into Kyn. To this purpose, Kyn concentrations were measured by high-performance liquid chromatography (HPLC) in 24 h-culture supernatants supplemented with 100 µM Trp in the last 8 h, as previously described [18].

### 2.6. Statistical Analyses

Data, expressed as the mean ± S.D., were analyzed by two-tailed unpaired Student’s *t*-test for two-sample comparison or paired Wilcoxon test in the skin test assays. For statistical analysis, GraphPad Prism 9.5.0 software for Windows (GraphPad Software, Boston, MA, USA) was used. A *p*-value ≤ 0.05 was considered statistically significant.

## 3. Results

### 3.1. Synthetic HIV-1–Derived Single-Stranded RNA-Oligonucleotides Confer Tolerogenic Activity on pDCs

**Figure 1:**
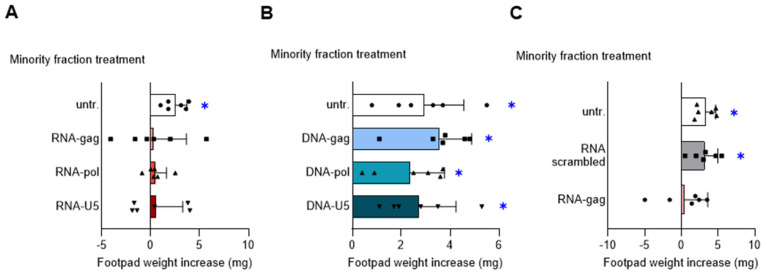
RNA-oligonucleotides based on the genomic sequence of HIV-1 confer tolerogenic properties to pDCs. Skin test reactivity of mice sensitized with splenic HY-pulsed immunostimulatory cDCs combined with a minority fraction (5%, indicated) of pDCs, left untreated (untr.) or stimulated with 1.5 μM of indicated RNA-oligonucleotides (**A**), DNA-oligonucleotides (**B**), or scrambled RNA-oligonucleotide as a control (**C**). Cells were i.p. transferred into syngeneic C57BL/6 recipient female mice to be assayed for footpad challenge with HY peptide 2 weeks after the immunization. Skin reactivity of the recipient mice (n = 6 per group) to the eliciting peptide is represented as the change in weight of the treated footpad vs. the vehicle-receiving counterpart. Data are reported as the mean value ± S.D. of three experiments. Significance is referred to as a paired Wilcoxon test (experimental vs. control footpads) in each group of mice; * *p* < 0.05.

Endosomal TLR-mediated detection of nucleic acids and production of type I IFNs are key elements of the antimicrobial defense strategy orchestrated by pDCs [19]. We previously demonstrated that synthetic CpG- and GpC-containing ODNs confer strong suppressive activity on pDCs by the engagement of TLR9 and TLR7, respectively, and the adaptor TRIF [5]. To determine whether HIV-derived oligonucleotides would be capable of shaping the immunomodulatory effects of pDCs, we first synthesized, based on the genomic sequence of HIV-1, the three phosphorothioate-protected single-stranded RNA-oligonucleotides RNA-gag, RNA-pol, and RNA-U5, and the three corresponding single-stranded DNA-oligonucleotides DNA-gag, DNA-pol, and DNA-U5 (Table 1). Then, we used these RNA- and DNA-oligonucleotides to condition the pDCs intended for a DTH skin test assay. In this experiment, according to a well-established protocol, the DC-operated induction of antigen-specific immunoreactivity vs. tolerance can be measured in vivo [18]. Female C57BL/6J mice were i.p. sensitized by immunogenic splenic CD8^−^ DCs (hereafter named cDCs), given alone or in combination with a pDCs fraction (5% of the final cell mixture), either untreated or treated with 1.5 μM RNA- (Figure 1A) or DNA-oligonucleotides (Figure 1B). For peptide loading, the final DC mixture was incubated with the HY peptide, a minor histocompatibility male antigen. Two weeks after priming, immune reactivity was assessed by intrafootpad challenge with the HY peptide. As expected, the priming ability of immunostimulatory cDCs was not affected by the presence of untreated pDCs. Interestingly, pretreatment of pDCs with RNA- (Figure 1A), but not with DNA- (Figure 1B), oligonucleotides negated the onset of HY-specific immunity, indicating that these RNA-oligonucleotides confer an in vivo detectable immunosuppressive phenotype on pDCs.

Since the tolerogenic effect was shared by the three HIV-derived RNA-oligonucleotides, we continued our study, focusing our investigation on RNA-gag only, reproducing a genomic sequence of an HIV-1 Gag protein, also in consideration that it promotes both IL-27 and IL-10 immunosuppressive cytokine by pDCs, differently from RNA-pol oligonucleotide inducing only IL-10 (Supplementary Figure S1). We repeated the skin test assay using the scrambled RNA-oligonucleotide reported in Table 1 as a specificity control for the observed RNA-gag tolerogenic effect. Since we found that scrambled RNA-oligonucleotide was ineffective in inhibiting the DTH response (Figure 1C), we confirmed the immunosuppressive activity of RNA-gag on pDCs involving IL-27 as demonstrated in a skin test assay including an anti-IL-27 treated group (Supplementary Figure S2). These data suggest that specific single-stranded RNA-, but not DNA-, oligonucleotides derived from HIV-1 can be sensed by pDCs and shape the immunomodulatory properties of these cells toward a tolerogenic phenotype.

### 3.2. The Tolerogenic Effect of RNA-Gag Requires TLR3 and TLR7

**Figure 2:**
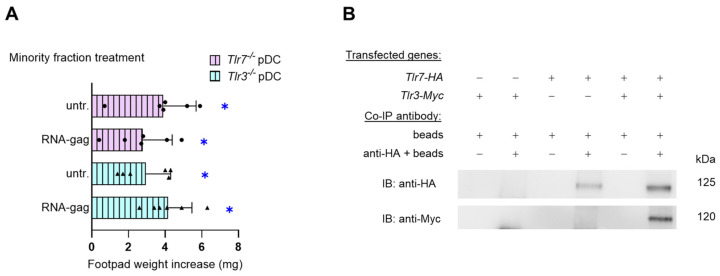
TLR3 and TLR7 are required for the acquisition of suppressive properties by pDCs upon RNA-gag oligonucleotide treatment. (**A**) Skin test reactivity of mice sensitized with a combination of splenic HY-pulsed immunostimulatory wild-type cDCs and a minority fraction (5%, indicated) of pDCs purified from either *Tlr7*^−/−^ (*Tlr7*^−/−^ pDC) or *Tlr3*^−/−^ (*Tlr3*^−/−^ pDC) mice. pDCs were left untreated (untr.) or stimulated with 1.5 μM RNA-gag before being HY-pulsed together with the cDC majority fraction, and i.p. transferred into syngeneic C57BL/6 recipient female mice. Two weeks after the immunization, mice were assayed by footpad challenge with HY peptide. Skin test reactivity of the recipient mice to the eliciting peptide (n = 6 per group) is represented as the change in weight of the treated footpad vs. the vehicle-receiving counterpart. Data are reported as the mean value ± S.D. of three experiments. Significance is referred to as a paired Wilcoxon test (experimental vs. control footpads) in each group of mice; * *p* < 0.05. (**B**) Co-immunoprecipitation assay in HEK cells transfected with *HA*-tagged *Tlr7* or/and *Myc*-tagged *Tlr3*. TLR7-HA protein complexes were immunoprecipitated with a specific anti-HA antibody and protein A-agarose (anti-HA + beads); samples immunoprecipitated with protein A-agarose alone were used as controls (beads). Membranes were immunoblotted with an anti-HA antibody as an immunoprecipitation control or with an anti-Myc antibody to reveal TLR7-HA/TLR3-Myc co-immunoprecipitated complexes.

Viral RNA molecules are commonly sensed by endosomal TLR7 and TLR8 (single-stranded RNA) [20] and TLR3 (double-stranded RNA) [21]. A common feature of both cytoplasmic and endosomal TLRs is that ligand binding usually occurs through interactions with a dimer of receptors, which can be homodimers, as in the case of TLR3, -4, -5, -7, -8, and -9, or heterodimers of TLR1/2 or TLR2/6 [22,23,24]. Thus, to verify whether, as presumably expected, the tolerogenic effects of synthetic RNA-gag required TLR7, we recurred to another skin test assay. To induce the skin test reactivity, we injected peptide-pulsed immunogenic cDCs admixed with a minority fraction of *Tlr7*^−/−^ pDCs, untreated or treated with 1.5 μM RNA-gag. Groups injected with a minority fraction of RNA-gag–treated or unstimulated *Tlr3*^−/−^ pDCs were also included as controls. Surprisingly, the immunosuppressive effect of RNA-gag, observed in Figure 1A,C, was negated not only in the absence of TLR7, as expected, but also when *Tlr3*^−/−^ pDCs were combined with immunogenic cDCs (Figure 2A), hinting at the involvement of both TLR7 and TLR3 in the sensing of this single-stranded RNA-oligonucleotide. To unveil the possible existence of a TLR3/TLR7 heterodimer, a complex combination never described before, the direct association of them was investigated by co-immunoprecipitation in HEK cells transfected with *HA*-tagged *Tlr7* and *Myc*-tagged *Tlr3* gene constructs (Figure 2B). As a loading control, whole cell lysate aliquots were saved before immunoprecipitation and analyzed by immunoblotting with anti-Myc, anti-HA, or anti-β tubulin antibodies (Supplementary Figure S3). Our data show that TLR3 forms heterodimers with TLR7 and suggest a previously unknown cooperation between the two endosomal TLRs, traditionally considered to be involved in the recognition of different types of RNAs (i.e., double-stranded by TLR3 and single-stranded by TLR7).

### 3.3. The RNA-Gag Triggers a Signaling Requiring TRIF, but Not MyD88, Adaptor

**Figure 3:**
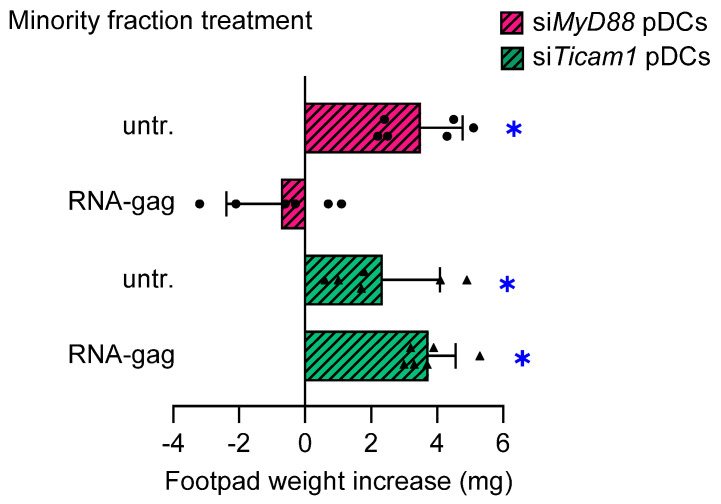
TRIF, but not MyD88, is required to transduce the immunosuppressive signal induced by RNA-gag in pDCs. The possible involvement of MyD88 and/or TRIF in signal transduction triggered by RNA-gag was investigated by the skin test. Mice to be assayed for skin test reactivity were sensitized with a HY-pulsed mixture of splenic cDCs, and a 5% minority fraction of pDCs having either MyD88 or TRIF silenced by siRNA (si*MyD88* pDCs and si*Ticam1* pDCs, respectively). pDCs were left untreated (untr.) or stimulated with 1.5 μM RNA-gag before being HY-pulsed with the cDC majority fraction, and i.p. transferred into syngeneic C57BL/6 recipient female mice. Two weeks after the immunization, the skin test reactivity of the recipient mice (n = 6 per group) to the eliciting HY peptide was recorded and represented as a change in weight of the treated footpad vs. the vehicle-receiving counterpart. Data are reported as the mean value ± S.D. of three experiments. Significance is referred to as a paired Wilcoxon test (experimental vs. control footpads) in each group of mice; * *p* < 0.05. One experiment is representative of three.

TLR signaling is broadly classified within MyD88- and TRIF-dependent pathways based on specific adaptor recruitment. Except for TLR3, all TLRs recruit MyD88 as a signaling adaptor. It is commonly believed that TLR4 is the only receptor using TRIF, TRAM, MyD88, and MAL, thus serving as a prototype for both the TRIF- and MyD88-dependent pathways [25]. Nevertheless, we previously demonstrated a possible involvement of TRIF adaptor in TLR7 signaling in pDCs [5]. To further dissect the signaling triggered by the RNA-gag, we set up a skin test assay with a combination of wild-type cDCs and pDCs, the latter transfected with siRNA to silence either TRIF or MyD88 (si*Ticam1* and si*Myd88* pDCs, respectively). Silencing efficiency was verified by end-point PCR (Supplementary Figure S4). In the absence of TRIF, RNA-gag failed to confer suppressive properties to si*Ticam* pDCs, while it was still effective on si*Myd88* pDCs (Figure 3), thus demonstrating that the TRIF adaptor alone is necessary to activate the TLR3/TLR7 signal transduction. Overall, these findings revealed the existence of a TLR3/TLR7/TRIF axis, confirming the ability shared by various TLRs to form different receptor clusters.

### 3.4. Arg1 Is Needed for the Tolerogenic Effect Induced by RNA-Gag on pDCs

**Figure 4:**
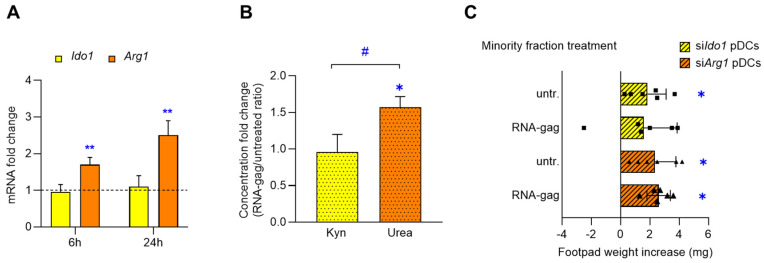
RNA-gag tolerogenic effect on pDCs involves *Arg1*, but not *Ido1*, induction and activity. (**A**–**C**) Splenic pDCs were exposed to RNA-gag (1.5 μM) or left untreated. (**A**) Real-time PCR analysis of *Ido1* and *Arg1* transcripts. For each time point of 6 and 24 h, data (means of three experiments using triplicate samples) represent the *Ido1* and *Arg1* transcript fold change in RNA-gag–treated relative to untreated pDCs, all normalized to the *Gapdh* expression. Dotted line, fold change = 1. Data are reported as mean value ± S.D. of three experiments. Student’s *t*-test (RNA-gag vs. untreated), ** *p* < 0.01. (**B**) Concentration fold change of Kyn and urea in culture supernatants and cell lysates, respectively, of RNA-gag–treated relative to untreated pDCs. Data are reported as mean value ± S.D. of three experiments. Student’s *t*-test (RNA-gag vs. untreated), * *p* < 0.05; RNA-gag/untreated ratio of Kyn vs. urea, # *p* < 0.05. (**C**) DTH skin test assay to assess the Arg1 involvement in the RNA-gag tolerogenic effects. Skin test reactivity of syngeneic C57BL/6 recipient female mice sensitized with a combination of splenic HY-pulsed immunostimulatory cDCs and a minority fraction (5%, indicated) of pDCs, silenced with either *Ido1* or *Arg1* siRNA (si*Ido1* pDCs and si*Arg1* pDCs, respectively), untreated (untr.) or stimulated with 1.5 μM RNA-gag. Two weeks after the immunization, skin test reactivity of the recipient mice (n = 6 per group) to the eliciting HY peptide was recorded and represented as a change in weight of the treated footpad vs. vehicle-receiving counterpart. Data are reported as mean value ± S.D. of three experiments. Significance is referred to a paired Wilcoxon test (experimental vs. control footpads) in each group of mice; * *p* < 0.05.

Dendritic cells exploit multiple mechanisms and signals to promote either immunity or tolerance toward specific antigens, depending on microenvironmental factors, such as the presence of cytokines or chemokines [26]. Ido1 and Arg1 are both immunoregulatory molecules expressed by DCs and can be independently activated or coactivated in response to specific stimuli, such as IFN-γ, TGF-β or IL-4 [9]. Therefore, we investigated the expression and catalytic activity of these enzymes in pDCs in response to the RNA-gag stimulation. As shown in Figure 4, while *Ido1* expression was similar in untreated and oligonucleotide-stimulated pDCs over time, *Arg1* mRNA was significantly increased by RNA-gag treatment at 6 h (1.7-folds, *p* = 0.0037) and 24 h (2.5-folds, *p* = 0.0029) (Figure 4A). The dosage of urea and Kyn—reflecting the catalytic activity of Arg1 and Ido1 enzymes, respectively—enlightened that augmented *Arg1* expression was accompanied by a corresponding increase in urea production in the RNA-gag–treated compared to the untreated pDC cell lysate (untreated 27.14 ± 1.2 µmol/mg of proteins, RNA-gag 42.61 ± 2.35 µmol/mg of proteins; fold change = 1.57, *p* = 0.0005), while the absence of *Ido1* induction was reflected by the unmodified Kyn concentration in pDC culture supernatant (untreated 31.3 ± 3.05 nM, RNA-gag 30.3 ± 5.03 nM; fold change = 0.96, *p* = 0.7833). Thus, the concentration-fold change plotted as RNA-gag/untreated ratio resulted in a significantly higher rate for urea than for Kyn (*p* = 0.0202) (Figure 4B).

To confirm the selective involvement of Arg1, rather than Ido1, in the suppressive mechanisms activated by RNA-gag in pDCs, either *Ido1* or *Arg1* were silenced by siRNA (si*Ido1* and si*Arg1* pDCs, respectively). Silenced pDCs (Supplementary Figure S4) were then assessed for their ability to sense RNA-gag and inhibit antigen-specific immune response in vivo. In DTH experiments with wild-type pDCs, RNA-gag treatment was able to prevent the immunogenic response otherwise observed in the untreated control group (Figure 1A and 1C). The silencing of Ido1 function did not modify such responsiveness to the skin test since RNA-gag–treated si*Ido1* pDCs inhibited the antigen presentation otherwise occurring in the untreated si*Ido1* pDCs control group (Figure 4C). On the contrary, *Arg1* silencing reverted the suppressive response triggered by RNA-gag (already observed in wild-type pDCs of Figure 1A and 1C), resulting in a significant footpad weight increase upon skin test challenge, similar to the untreated si*Arg1* pDCs control group (Figure 4C). Thus, our data demonstrate that RNA-gag is effective in increasing protein expression and enzymatic activity of Arg1 and requires the expression of *Arg1*, but not *Ido1*, for the induction of a suppressive phenotype in pDCs.

## 4. Discussion

The mammalian innate immune response is active in the early stages of defense against invading pathogens. One of the primary receptor families sensing pathogens’ molecular structures and triggering the innate immune response is that of the TLRs, comprising ten functional receptors in humans (TLR1 to -10) and twelve in mice (TLR1 to -9 and TLR11 to -13). TLRs play a pivotal role both as direct activators of prompt innate immune responses and as linkers for the recruitment of signaling molecules that start adaptive immunity. The triggering of such an immune response efficiently occurs in DCs, secreting cytokines and driving antigen presentation to T-cells. Among DCs, the subset of pDCs expressing the specific receptors TLR7 and TLR9 is particularly specialized in the sensing of pathogens’ nucleic acids [27,28]. As mammals have a limited number of innate immune receptors to recognize a potentially unlimited number of microbial components (commonly defined as pathogen-associated molecular patterns, PAMPs), these receptors are endowed with extensive flexibility and form stable homo- or heterodimeric complexes with a huge variety of molecules, ranging from hydrophilic nucleic acids to hydrophobic lipids. For example, TLR4, the prototypic receptor for LPS, though traditionally known to be active as a homodimer [29], forms functional heterodimers with TLR6 and TLR2. Interestingly, the latter association occurs upon recognition of the HIV-1 gp120 protein [30]. Similarly, TLR5, the flagellin receptor, has been recently demonstrated to be capable of forming a heterodimer with TLR4 in response to LPS, thus modulating the canonical TLR4 signaling (involving both MyD88 and TRIF) by specifically promoting the activation of the MyD88 pathway [31]. These findings on the flexibility adopted by TLR family members, in combining each other, are in line with our data suggesting the occurrence of a functional TLR3/TLR7 complex in pDCs, capable of recognizing the HIV-1–derived single-stranded RNA-gag oligonucleotide and activating a peculiar signaling pathway via TRIF recruitment. In general, we could speculate that other yet unidentified functional pairs of TLR heterodimers might exist, a possibility underscoring the complexity and versatility of this receptor family. Our discovery of a possible TLR3/TLR7 association in pDC is reinforced by the knowledge that these two receptors share the same intracellular localization [32], both recognize viral RNAs and are required for mounting anti-viral innate immune responses.

Exploration of the molecular basis of HIV-1 innate immune sensing is still a topic of intense interest. To date, several families of pattern recognition receptors (PRRs) have been indicated as sensors able to detect HIV-1 infection (e.g., TLRs, RIG-I-like receptors (RLRs), and cytosolic DNA sensors) [11,20,33]. Both RLR and TLR family members (specifically TLR7 and TLR8) have been characterized as PRRs of HIV-1 RNAs [20]. It has been demonstrated that TLR7 detects HIV-1 single-stranded RNA in pDCs after endocytosis of the virus [34] and preferentially recognizes the guanosine (G)- and uridine (U)-rich single-stranded RNA oligonucleotides derived from the virus to initiate the production of proinflammatory cytokines and chemokines [20]. Although the role of pDCs in the immune response after HIV-1 sensing is still not clear, for these cells, a pivotal role in the general state of immunosuppression after the viral infection has been postulated. In fact, pDCs have been demonstrated to be capable of inciting the recruitment of CCR5^+^ CD4^+^ T-cells to mucosal sites of HIV-1 inoculation during transmission [35] and inducing apoptosis of CD4^+^ T-cells through their persistent production of type I IFNs [12]. Moreover, pDCs can favor the generation of immunosuppressive regulatory T-cells over immunostimulatory Th17 cells, a mechanism including the expression of Ido1 or other immunoregulatory molecules such as Arg1 [7].

Thus, besides antiretroviral therapies limiting virus replication, novel methods are needed to strengthen the immune system, overcome the state of immune tolerance induced by HIV-1, and reactivate an efficient immune response toward the virus. The finding that the functional interaction between TLR3 and TLR7 results in the acquisition of tolerogenic properties by pDCs after HIV-1–derived single-stranded RNA sensing could suggest investigating a possible novel class of drugs interfering with this specific molecular mechanism, aiming to restore a proper and efficient anti-viral immune response. Over the past years, many efforts have been made to find drugs that could be therapeutically beneficial during retroviral infection by acting as TLR agonists [36] by enhancing the adaptive immune-mediated clearance of the virus. As an example, agonists for TLR3, TLR7, TLR8, and TLR9 have been shown to be capable of inhibiting HIV-1 infection and induced IFN-α-stimulated gene expression in PBMCs in vitro [37]. Moreover, Jimenez-Leon et al. recently demonstrated that TLR7 and TLR9-specific pDCs stimulation induces a T-cell mediated antiviral response, which is essential for HIV-1 eradication strategies [38]. Counterbalancing this hypothesis, HIV-1 often exhibits enhanced replication in an inflammatory environment [39], and activated T-cells are key target cells for viral infection and spreading [40]. Finally, yet importantly, as we demonstrated in the present study, stimulation of TLRs with viral components can lead to a state of immune inactivation rather than immune stimulation. Thus, the pharmacological stimulation of TLRs in the context of HIV-1 infection might enhance HIV-1 replication and/or pathogenesis. In this scenario, the discovery that Arg1, an enzyme contributing to an immunosuppressive microenvironment in many cancers [41], can mediate the tolerogenic signaling triggered upon TLR3/TLR7 stimulation could open new avenues of research to find innovative immunostimulatory drugs inhibiting Arg 1 for improving antitumor immunity in cancer immunotherapy [41]. In the progression of AIDS, immune aging is a significant feature of chronic HIV-1 infection being associated with non-AIDS-related events; explained in more detail, neutrophil aging is closely related to T-cell exhaustion shown in HIV-1 infected patients. Immunosuppressive aged neutrophils from HIV-diagnosed, treatment-naïve patients have high *PDL1* and *ARG1* expression; moreover, Arg1 blocking partially reversed the immunosuppression of CD8^+^ T-cells by neutrophils [42]. Thus, this evidence demonstrates that targeting Arg1 at multiple levels could represent an efficient approach to recovering T-cell dysfunction in HIV-1-infected patients.

The occurrence of signaling triggered by HIV-1–derived single-stranded RNA molecules via the formation of a TLR3/TLR7 heterodimer certainly needs to be further elucidated using more extensive experimental settings (i.e., in human pDCs from patients infected by HIV-1). However, our findings represent an important suggestion that pDCs are a valuable tool for direct immune responses toward a state of tolerance. These cells are characterized by functional flexibility in activating but also suppressing both inflammatory/innate responses and adaptive immunity [7,43]. In murine experimental models, they represent a precious tool for dissecting the potential of certain stimuli, including synthetic GpC-ODNs, to induce tolerogenic responses [5,7]. Over the past few years, in vitro-generated tolerogenic dendritic cells (tol-DCs) with immunoregulatory functions have attracted much attention for their important protective role in the control of undesired and pathologic immune responses in organ transplantation, autoimmune diseases, and allergy [44,45,46]. Currently, though patients can successfully acquire immunological tolerance by treatment with non-specific immunosuppressive agents, these drugs often cause serious side effects, such as opportunistic infections, according to most clinicians. Recent studies have focused on the prospective value of tol-DCs to induce clinical organ graft-specific tolerance and prolong graft survival [47], as well as be used for clinical application in autoimmune diseases, such as multiple sclerosis [48], and in the immunotherapy of allergies [49].

## 5. Conclusions

In conclusion, our finding revealed that novel, synthetic oligonucleotide shapes the immunomodulatory properties of pDCs via a TLR3/TLR7/TRIF-mediated pathway and Arg1 increased activity, rendering them, de facto, tol-DCs. These observations could represent a useful starting point for the tuning of innovative protocols generating promising “negative cellular vaccines” that are useful in inducing a protective state of tolerance against transplant rejection, autoimmune diseases, and allergies.

## Figures and Tables

**Table 1:** RNA- and DNA-oligonucleotides based on the genomic sequence of human HIV-1.

Oligonucleotides	Sequence (5′-3′)
RNA-gag	UUG UUA AGU GUU UCA AUU GU
RNA-pol	CAU AUU UUU CAG UUC CCU UA
RNA-U5	GCC CGU CUG UUG UGU GAC UC
RNA scrambled	GGU UUU AAU UUU GGU UUA AC
DNA-gag	TTG TTA AGT GTT TCA ATT GT
DNA-pol	CAT ATT TTT CAG TTC CCT TA
DNA-U5	GCC CGT CTG TTG TGT GAC TC

## Data Availability

The original contributions presented in the study are included in the article/Supplementary Materials; further inquiries can be directed to the corresponding author.

## Supplementary Materials

The following supporting information can be downloaded at: https://www.mdpi.com/article/10.3390/cells13131088/s1, Figure S1: Cytokine in the supernatants of RNA-gag or RNA-pol treated pDCs; Figure S2: Involvement of IL-27 in the suppression of skin test assay reactivity; Figure S3: Loading control of co-immunoprecipitation assay in HEK cells transfected with *HA*-tagged *Tlr7* or/and *Myc*-tagged *Tlr3*; Figure S4: Semi-quantitative evaluation of siRNA silencing by end-point PCR.
