# Supplementary material for: Sensing of an HIV-1–Derived Single-Stranded RNA-Oligonucleotide Induces Arginase 1-Mediated Tolerance
Source: Cells. 2024 Jun 23;13(13):1088. doi: 10.3390/cells13131088 (PMC11240372; doi:10.3390/cells13131088)
Supplement: Supplementary file 1 [file cells-13-01088-s001.zip › cells-3027165-supplementary.pdf]

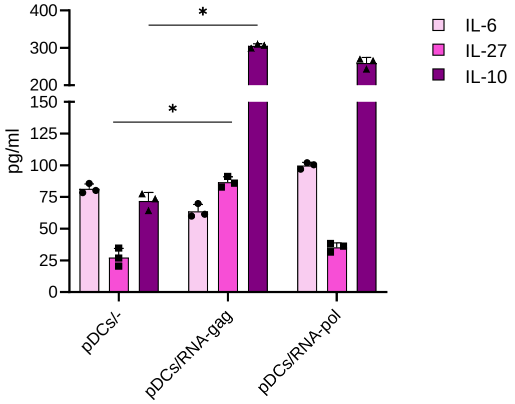

Figure S1. Cytokine in the supernatants of RNA-gag or RNA-pol treated pDCs. Supernatants of the pDCs prepared for the skin test assay (overnight incubation with 1.5 μM RNA-gag or RNA-pol oligonucleotides, or left untreated) were recovered for ELISA cytokines analysis (IL-6, IL-27, and IL-10). For cytokine statistic analysis, the nonparametric Kruskal-Wallis test was used (comparison of treated vs. untreated pDCs); *$P < 0.05$.

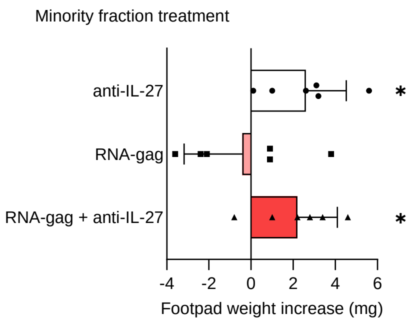

Figure S2. Involvement of IL-27 in the suppression of skin test assay reactivity. HY-pulsed wild-type CD8- DCs, in combination with a minority fraction of pDCs, treated in vitro overnight with 5 μg/ml anti-IL-27 (goat anti-mouse IL-27 p28 antibody, R&D Systems, Minneapolis, MN, USA) 1.5 μM RNA-gag, or both, were injected in recipient mice. Skin reactivity to the eliciting peptide was recorded at 15 days and plotted as a change in footpad weight. The data are reported as the mean value ± S.D. of three experiments. Significance is referred to as a two-tailed paired Wilcoxon test (experimental vs. control footpads) in each group of mice; *P < 0.05.

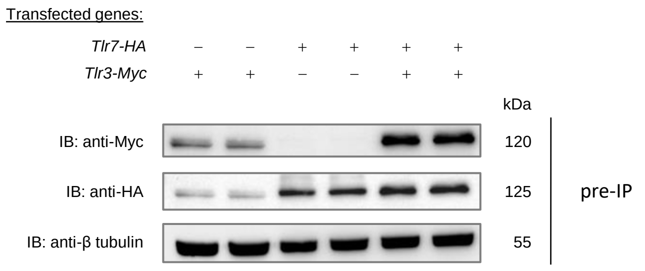

Figure S3 Loading control of co-immunoprecipitation assay in HEK cells transfected with *HA*-tagged *Tlr7* or/and *Myc*-tagged *Tlr3*. Whole cell lysates aliquots were saved before immunoprecipitation and loaded as pre-IP controls. The membrane was sequentially immunoblotted with anti-Myc (9E10 clone, Origene, Rockville, MD, USA), anti-HA (2-2.2.14 clone, Thermo Fisher Scientific, MA, USA) and anti-β tubulin (AA2 clone, Merk-Sigma Aldrich, Darmstadt, Germany) antibodies. Anti-Myc, anti-HA, and anti-β tubulin share the same secondary antibody (HRP conjugate goat anti-mouse IgG antibody, Merk Millipore, Burlington, MA, USA)

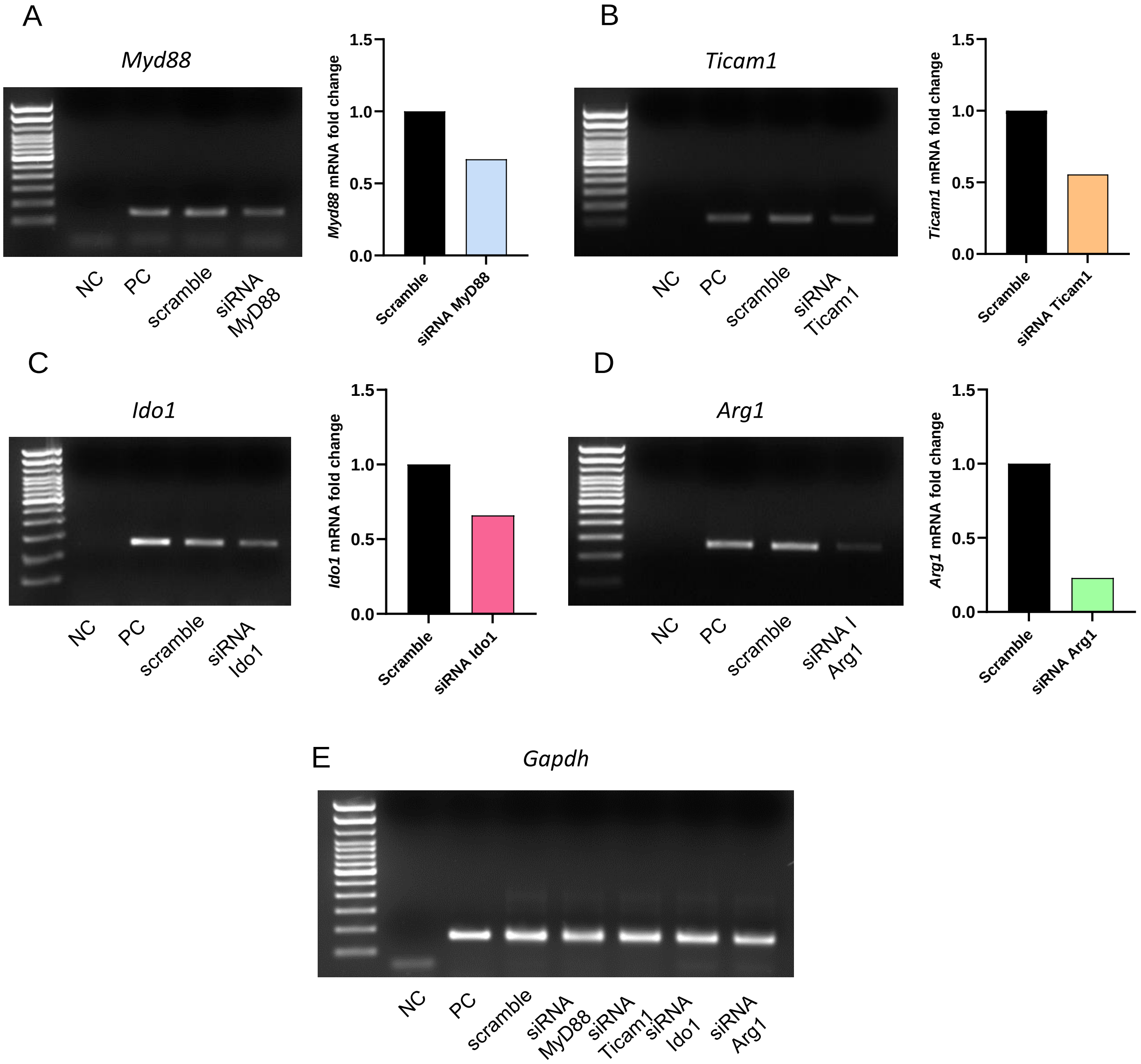

Figure S4. Semi-quantitative evaluation of siRNA silencing by end-point PCR. A-E: cDNAs from siRNA- or scramble-transfected pDC were amplifyed with specific primers (annealing at 60°C, 40 cycles). NC, negative control; PC, positive control. Colored histograms represent the *Myd88*, *Ticam1*, *Ido1* and *Arg1* transcript fold change in pDCs transfected with respective siRNA (siRNA- vs. scramble-transfected), while black histograms represent the scramble control (fold change = 1), all normalized to the *Gapdh* expression. For *Ido1* and *Arg1* primers, see materials and methods section; *Myd88* forward primer 5′-CTGGCCTTGTTAGACCGTGA-3′; *Myd88* reverse primer 5′-GTGGGACACTGCTTTCCACT-3′; *Ticam1* forward primer 5′-GAGGCAGGACTGTGTGATCC-3′; *Ticam1* reverse primer 5′-TTGGGGAGTGTTCATCCAGC-3′.
